# Trends in involuntary admissions for observation in malta

**DOI:** 10.1192/j.eurpsy.2021.970

**Published:** 2021-08-13

**Authors:** M.A. Zammit, M. Agius, J. Cutajar, B. Micallef Trigona

**Affiliations:** 1 Acute Psychiatry, Mount Carmel Hospital, Attard, Malta; 2 Complex Psychiatry, Mount Carmel Hospital, Attard, Malta

**Keywords:** Mental Health Act, psychosis, Involuntary Admission

## Abstract

**Introduction:**

Schedule II of the 2013 Mental Health Act is part of the legal framework for involuntary admission to a licensed mental healthcare facility in Malta (Mount Carmel Hospital) for observation.

**Objectives:**

To identify trends in presenting features cited by registered specialists in psychiatry in Schedule II applications as well as impact of time of day on involuntary admission.

**Methods:**

Schedule II forms relating to all involuntary admissions to Mount Carmel Hospital between 01 June 2018 and 01 June 2019 were retrieved from paper files (n=364). Details relating to reason for using this legal framework were recorded and processed through custom linguistic analysis. Timings of application were also assessed. Data Protection permissions to retrospectively access patient files were obtained. All data collected was de-identified at source.

**Results:**

The commonest reason for use of Schedule II was psychosis (n=139). Substance abuse was recorded in 68 cases, with alcohol and cannabinoids the commonest substances cited. 155 instances relate to situations of increased risk, the commonest being aggressive behaviour (n=74). 61 cases recorded suicidal intent. Peak use of this schedule occurs between 17:00 and 18:00, which is outside normal working hours.

**Conclusions:**

Predominance of psychosis as a reason for involuntary admission concurs with trends reported internationally, including recent German, Irish and Dutch reports, as does increased use of involuntary admission with out-of-hours presentations. Practices relating to involuntary admission to a mental healthcare facility in Malta appear to reflect general trends in other European cohorts, despite differing legal frameworks.

